# A Clinical Lecture on Flat-Foot

**Published:** 1906-12-01

**Authors:** William Billington

**Affiliations:** Senior Surgeon to Out-patients, Queen's Hospital, Birmingham


### A Clinical Lecture ON FLAT-FOOT.

By WILLIAM BILLINGTON, M.B., M.S.(Lond.), F.R.C.S., Senior Surgeon to Out-patients, Queen's Hospital, Birmingham.

Gentlemen,—No apology is needed for my choice of a subject to-day. Sufferers from flat feet are very common amongst hospital out-patients, and their successful treatment is tedious and often disappointing. I well remember a distinguished surgeon upon the staff of this hospital saying that he always felt inclined to run away when confronted with one of these cases.

The term "flat-foot" is sometimes used to include true cases of talipes valgus, in which the sole is everted. This latter deformity is congenital, or induced by the unbalanced action of the peronei muscles, and has little in common with the condition to which I am about to refer. The term should be reserved for those cases where the instep is unduly low from giving way of the longitudinal arch of the foot.

### Etiology of Flat-foot.

The deformity is always due to yielding of the longitudinal arch of the foot under the weight of the body. Hence anything that tends to weaken the arch or to throw additional strain upon it, may be a cause of flat-foot. The condition may develop quickly with acute symptoms, or more gradually.

Most of the cases of flat-foot that come under observation are adolescents between 16 and 20 years of age. There are several reasons for this. At puberty the body weight increases rapidly, and at the same time there is often a change of occupation involving long hours of standing, as in the case of barmaids, shop assistants, nurses, etc., amongst which classes flat-foot is most common. It thus follows that a greater strain is thrown upon the arches of the feet for a longer time. In addition, it frequently happens that increased weight is not balanced by increased muscular power. Many of these people are anæmic, their general health is defective, and their muscles flabby and ill-developed. The arches are not strengthened sufficiently to enable them to sustain the extra strain, and they give way under it. Normally, the tendons passing into the sole from the back of the leg play an important part in bracing up the arch, but when the corresponding muscles are weak and easily tired this support is largely wanting.

Whether the affection develops quickly or slowly depends largely upon the conduct of the patient after the appearance of the initial symptoms. If, in spite of aching of the feet and a sense of tiredness in the legs, he persists in his occupation and neglects to rest, the condition becomes rapidly worse and the symptoms more severe. If, however, the strain is less continuous, the arches give way much more gradually, and it may be that discomfort is only felt after an unusual amount of standing or walking, or when the general health is below normal.

The following histories are typical:—

A. T., aged 18, recently employed as a billiard marker, complained of severe aching pain in his feet when standing. It was so severe that he had to give up his employment. He had to stand for 14 hours a day in an underground room, and had few opportunities of resting. His feet began to be painful within a few weeks of commencing work, and his general health suffered from the close confinement. He continued his work, however, in spite of the fact that the pain in his feet rapidly got worse. Ultimately it became so severe that he was almost crippled, and could only hobble about with great difficulty. After sitting down for a few minutes, the pain on getting up again was so acute that he had to take hold of something to prevent himself from falling. At this time he noticed that his ankles and feet were much swollen at night. When seen, there was some fullness behind each malleolus and on the dorsum of the foot. At rest, the arches of the feet appeared nearly normal, but on getting him to stand up they almost entirely disappeared, the inner margin of the foot coming in contact with the floor. At the same time the foot was abducted and the sole everted. The patient complained of much tenderness when firm upward pressure was made over the head of the astragalus.

In this case the symptoms were acute because the patient neglected to rest until compelled to do so.

Miss N., aged 23, commenced work as a hospital nurse some four months ago, and was obliged to be on her feet for many hours consecutively every day. Six weeks after commencing her duties she began to have trouble with her feet. She experienced aching pain in the centre of the sole, and complained of a "tired feeling" in her legs. The symptoms increased in severity, and at the end of three months she was compelled to resign. She thought that the symptoms were partly caused by the wearing of slippers with very low heels while on duty. When seen, she had been at home for a month, doing only light housework and resting whenever she felt tired. The symptoms had almost entirely disappeared, and she merely sought advice as to the possibility of a return to nursing. On examination the arches of the feet were very low, but little alteration took place when she put her weight upon them. There was no swelling and no tenderness on pressure. Evidently her feet were able to withstand an ordinary amount of strain, and only when this was excessive or too prolonged did symptoms of flat-foot develop.

Trauma is an important etiological factor in flat-foot, though insufficient stress is laid upon it in text-books. It is well known that flat-foot frequently follows Potts' fracture, and is then due to imperfect reduction of the deformity at the time of injury, or to subsequent eversion of the sole when the patient is allowed to get about too soon without efficient support for the leg and foot. I think, however, that it is not so well known how frequently flat-foot develops after severe crushes, such as are caused by the wheel of a cart passing across the dorsum, or a heavy weight falling upon it. In these cases the ligaments are severely strained, and, becoming softened by the resulting inflammation, are apt to yield under the weight of the body and allow the arch to descend. I now, as a routine procedure, apply a firm pad of lint to the instep to support the arch from the first after any severe injury to the foot. This pad is worn for several weeks after the patient has commenced to get about, only being discarded when it is clear that all inflammation has subsided and the ligaments have regained their normal strength. This is the more necessary on account of the fact that flat-foot as a sequela of injury is especially troublesome to treat effectively. There is usually a good deal of inflammatory thickening in the sole and much tenderness, so that supports for the arch are badly tolerated. Later, the deformity is difficult to reduce, on account of the dense adhesions which are apt to form in and around the joints.

The following is an illustrative case:—

T. W., aged 25, sustained a severe injury to his right foot six months ago. The wheel of a heavy cart passed across the dorsum. When seen the foot was greatly swollen, and for several weeks he was unable to bear any weight upon it. Moulded splints were applied, but no special precautions were taken to maintain the integrity of the arch, and he was allowed to get about, when able to do so, without the instep being supported. The man now seeks relief from pain in his foot, which is so severe that he cannot follow his employment. He is a tram-driver, and has to stand for many hours at a time. His difficulty is increased by the fact that he has to sound the alarm bell by stamping on a metal plate with the injured foot. The pain has increased in severity, and has compelled him to cease work altogether. On looking at the foot you will see that the instep is quite flat, and that the inner margin of the sole is convex instead of concave. Beneath the head of the astragalus is a dense mass of inflammatory tissue, which is very tender on firm pressure.

Acute flat-foot is always caused by some inflammation affecting the tarsal joints and the surrounding structures. It occurs in gonorrhœal rheumatism and occasionally in rheumatism itself. The arch sinks in consequence of inflammatory softening of the supporting ligaments. When the condition is due to gonorrhœa many adhesions are apt to form and give rise to considerable difficulty in the reduction of the deformity.

Occasionally, also, flat-foot is secondary to other deformities, particularly genuvalgum, the weight of the body acting exclusively through the inner half of the foot and causing the longitudinal arch to become flattened. It also occurs in association with rickets.

The following case is of interest as showing how the symptoms of flat-foot may first appear after unusually severe exercise, such as mountain-climbing or prolonged walking. Probably there is always a previous tendency to the condition, but no trouble is experienced until the feet are subjected to some unusually severe strain.

Mr. K., aged 21, came complaining of aching in the feet and legs after an unusual amount of walking or prolonged standing. He stated that a year previously he played in several football matches on successive days, the ground being heavy and sodden with rain, and the physical strain unusually severe. Previous to this he had never noticed anything wrong with his feet, and led an active life, which involved much standing and walking. For some days afterwards his legs and feet ached severely, and enlarged and tender glands appeared in the femoral regions. He returned to work after a week's rest, but could only get about with difficulty for several weeks. Since then any unusual amount of walking or standing causes a return of the symptoms, and he had been compelled to forego all active exercise. On examination the feet were large, and the inner borders unusually long. The arches were low, and when the body weight was put upon them became almost obliterated.

The excessive strain a year ago probably caused some inflammatory softening of the ligaments, with consequent yielding of the arch under the weight of the body. Had the patient rested longer, or had the arches been properly supported, the deformity would probably not have occurred.

### Pathological Changes.

The appearance of the foot is quite characteristic. The inner border looks unusually long, and inquiry often elicits the information that the ordinary boot appears to be too short, the great toe being forced against the end of it. The instep is less marked than usual, and on standing the arch disappears altogether, while the whole foot is seen to roll outwards, so that the inner margin comes in contact with the ground in its whole extent. That this is the case is made obvious by getting the patient to dip his feet in whitewash and then to walk over a brown-paper sheet. The footprints show a continuous line along the inner border in place of the normal interrupted one. After standing some time the veins on the dorsum are seen to be engorged with blood, and there is some puffiness around the malleoli.

In order to clearly understand the morbid changes that occur in flat-foot it is essential to recall the normal structure of the longitudinal arch and the mechanism by which it is maintained. The arch consists of two pillars and a keystone. The posterior pillar is formed by the os calcis, and is the shorter and stronger of the two. The anterior pillar, longer and more slender, is formed by the scaphoid, cuneiform bones, and the three inner metatarsals. Between the two pillars, and forming the keystone of the arch, is the head of the astragalus. All the structures in the sole which pass between the two pillars assist in supporting the arch. Of these, the most important is the inferior calcaneo-navicular ligament, passing from the sustentaculum tali of the os calcis to the under surface of the scaphoid bone. This ligament is a broad, dense, somewhat elastic band, and the head of the astragalus, which rests directly upon its upper surface, can only descend when the ligament is overstretched. Of the accessory supporting structures, the most important is the tendon of the tibialis posticus. This strong tendon passes into the sole behind the internal malleolus, and is inserted primarily into the tubercle of the scaphoid. From this point fibres extend in all directions, and make attachments to all the tarsal and metatarsal bones, with the single exception of the astragalus. The tendon and its ligamentous prolongations help considerably to brace together the various bones which enter into the formation of the longitudinal arch. Weakness of the tibialis posticus muscle, with consequent diminution of the support it normally gives to the arch, is a not unimportant factor in the production of flat-foot. The tibialis anticus muscle and tendon, tending as they do to prevent eversion of the sole, also help to maintain the arch of the instep.

In flat-foot the pillars of the longitudinal arch become separated and the keystone descends. The head of the astragalus becomes partially dislocated downwards and inwards from the scaphoid, and in bad cases may only articulate with the latter bone at its extreme outer end. It comes to form a marked prominence on the inner border of the foot, and can readily be felt beneath the skin. The inner half of the sole is applied flat to the ground, and the inner margin of the foot is elongated, and may be convex instead of concave. At the same time the whole foot is abducted, and the external malleolus is much less prominent than usual, being buried, as it were, by the structures on the outer side.

Associated with flat-foot it is very common to find more or less rigidity of the first metatarso-phalangeal joint. This is in part caused by the great toe being driven against the end of the boot, as a result of the increase in length of the inner side of the foot that occurs when the arch sinks.

Descent of the longitudinal arch causes loss of elasticity in the foot, so that shocks and jars are communicated more forcibly to the legs and trunk. The normal protection which is afforded to the soft structures in the sole is also removed, and these may be injuriously compressed between the boot and the tarsal bones. This gives rise to pain and impedes circulation. The symptoms complained of are caused by (1) the loss of elasticity; (2) the strain upon weakened and overstretched ligaments; and (3) direct pressure upon the nerves and vessels in the sole.

### Symptoms.

The symptoms of flat-foot vary greatly in severity, but are always very similar in kind. They may be so acute that the patient is completely crippled, or occur only when unusual strain has been thrown upon the feet, or the general health is impaired.

Pain is a constant feature. It is usually dull and aching in character, and steadily increases in severity towards night. The pain is usually referred to the centre of the arch, but is very common at the sides of the ankle.

The pain is generally stated to be more severe on the outer side of the ankle, and to be due to direct pressure of the external malleolus upon the structures on the outside of the foot. In my experience, however, pain is equally common on the inside, radiating from the instep behind the malleolus and into the leg. That the pain is mainly caused by the strain thrown upon the structures in the sole by the weight of the body is evidenced by the fact that it is at once relieved by lying down, and is usually much less severe when the arch is supported by a pad or special sock.

Tenderness is variable. Its presence may be taken as an indication of inflammation of the ligaments of the sole. It is most marked in acute cases and those of traumatic origin, and may completely cripple the patient. It is most readily demonstrated by making firm upward pressure from the sole towards the head of the astragalus. The presence of tenderness introduces difficulties in the way of treatment, the patient being unable to bear the pressure of mechanical supports.

More or less swelling of the dorsum of the foot and around the ankle is common in bad cases. The swelling appears after walking or standing, and is worse towards evening, usually disappearing after a night's rest. In a few cases this swelling is of inflammatory origin, but more usually it seems to be due to mechanical interference with the circulation of the foot. Venous return is obstructed in the sole and the blood is forced into the veins of the dorsum, which may be seen to become distended when the patient stands up.

### Treatment.

The results of treatment in flat-foot are not altogether satisfactory, and disappointment to the patient is common. This is due to the fact that he is generally unwilling to forego the occupation or exercise which has produced the trouble. A great deal can be done, however, to relieve the symptoms in all cases, and in many a cure is possible.

I have already referred to the importance of preventive treatment in cases of trauma, and would again emphasise the necessity for efficient support to the arch until all inflammatory reaction has subsided.

In acute cases rest is essential. All strain must be taken off the arches until the acute symptoms have subsided. After a few days in bed a plaster case should be applied from the roots of the toes to a point well above the ankle. The case is applied with the deformity over-corrected. After five or six weeks the case may be removed and the foot examined. If all tenderness has disappeared, friction and massage are necessary to overcome the tendency to stiffness, and the arch is suitably supported as described below. If rheumatism or gonorrhœa exist, they must receive appropriate treatment.

The treatment of the ordinary form of chronic flat-foot must proceed along definite lines. In all cases the indications are to restore the arch, to diminish the strain upon it, and to supplement and strengthen as far as possible the structures which support it.

In most cases it is not difficult to restore the arch to its normal state by simple manipulation. In others this is impossible, and more radical measures are necessary. I shall confine my remarks to the treatment of those cases in which it is possible by simple manipulation, or by forcible wrenching under an anæsthetic, to correct the deformity.

The history of each case must be carefully gone into. The nature of the patient's occupation ascertained, his general health investigated, and any condition likely to induce general debility sought for. In this way it is often possible to glean valuable information as to the cause of the condition. Attention to the general health is of great importance. These patients are often debilitated, with flabby muscles and impoverished blood, and it must be borne in mind that the symptoms become aggravated when the patient is tired and run down.

The arch having been restored, by forcible wrenching under anæsthesia, if necessary, and the subsequent application of a plaster case until all inflammatory symptoms have subsided, it must be supported by mechanical means. The Whitman's instep sock is the simplest and best form of support. It consists of an aluminium or steel spring covered with wash-leather or some other suitable material. The sock fits the hollow of the instep, and the spring comes into action when the arch descends unduly. It is essential that each case should be specially fitted, or much discomfort will result. The sock is worn inside the boot, the instep of which should also be strengthened to further support the arch of the foot.

If it can be borne, the Whitman spring gives great relief, but the pressure cannot always be tolerated. To find out whether it can be worn, I first of all apply a woollen pad for a week or two. The pad consists simply of cotton wool covered by lint, and is made to accurately fit the instep, being kept in place by a few turns of bandage. If this pad gives relief Whitman's sock is ordered, but if the pressure of the pad causes discomfort it is perfectly certain that the sock could not be worn.

The surgeon should not be satisfied with providing against descent of the arch by mechanical supports only. Attempts should also be made to strengthen it. If kept in the natural position, the over-stretched ligaments tend of themselves to contract and regain their strength. In addition, the muscles of the sole and those of the leg can be strengthened by massage and exercise. I always order the foot and leg to be well rubbed with some stimulating liniment for 10 or 15 minutes every night. In the morning the feet and legs are douched with cold water and well rubbed with a towel. The most useful exercise is that of raising the heels and standing on the toes. It should be carried out with the feet bare, or in stockings only. The feet are placed together, and the patient raises himself on his toes several times in succession. At first this can only be done once or twice at a time, but with practice the exercise is performed more easily and for a longer time.

Finally the patient should be warned not to overtire the feet. Aching is an indication for rest, and when it occurs the weight of the body should be taken off the feet.

